# Post-endodontic pain following root canal treatment in permanent teeth among Indian paediatric patients

**DOI:** 10.6026/973206300200571

**Published:** 2024-05-31

**Authors:** Sajid Khan, Arunendra Singh Chauhan, Khushtar Haider, Saima Ali, Parul Shakarwal, Akriti Chauhan

**Affiliations:** 1 Department of Pedodontics and Preventive Dentistry, Career Dental College, Lucknow, Uttar Pradesh, India; 2 Conservative Dentistry and Endodontics, Inderprastha Dental college, Ghaziabad, Uttar Pradesh, India; 3 Department of Public Health Dentistry, Maharishi Markandeshwar College of Dental Sciences and Research, Mullana, Haryana, India; 4 Department of Conservative Dentistry and Endodontics, Sri Sukhmani Dental College and Hospital, Derabassi, Punjab, India; 5 Department of Public Health Dentistry, Maharishi Markandeshwar College of Dental Sciences and Research, Mullana, Haryana, India; 6 Department of Pedodontics and Preventive Dentistry, Seema Dental College and Hospital, Rishikesh, Uttarakand, India

**Keywords:** Incidence, post endodontic treatment pain, permanent teeth, paediatric patients

## Abstract

Pulp status in permanent teeth and post endodontic pain (PEP) has not been assessed properly in pediatric patients. Therefore, it is of interest to assess the prevalence, severity of PEP in permanent teeth after root canal therapy and retreatment in paediatric patients. Hence, 127 pediatric patients who had root canal therapy (RCT) for permanent teeth with necrotic pulp, vital pulporendodonticre treatment were considered. Assessment of incidence intensity of PEP at 6 hours and 18 hours after therapy was completed. The incidence and intensity of PEP in permanent teeth in paediatric patients was greater in teeth with vital pulp. It was low in teeth with necrotic pulp. The incidence of spontaneous PEP was greater in all treatment groups as compared to stimulated PEP at 6 hours after treatment. Thus, root canal therapy of teeth with viable pulp produced a noticeably greater incidence and intensity of PEP in permanent teeth in paediatric patients.

## Background:

An essential component of endodontic therapy is the prevention and control of post endodontic pain (PEP) [1, 2-3]. Patients' perceptions of upcoming dental procedures can be improved, their pain tolerance can be raised, and the trust they have in their dentists can be bolstered by educating them about predicted PEP and providing drugs to control it [4, 5, 6-7]. There has been research on the connection between the frequency and severity of flare-ups and the health of the restored teeth, but the findings have been mixed [8, 9, 10-11]. According to study, flare-ups occurred more frequently in non-vital teeth following retreatment as well as endodontic treatment in comparison to vital teeth. Another study noted that tooth vitality had no bearing on the frequency or severity of flare-ups [12, 13, 14, 15-16]. Pulp status and any PEP have not been observed to be correlated. After endodontic treatment, PEP (not just flare-ups) is highly common, and over 50% of patients who suffer PEP report having significant pain [17, 18-19]. However, no research has assessed the prevalence and seriousness of PEP in teeth with viable or necrotic pulp following initial RCT and subsequent retreatment [20, 21, 22- 23]. Therefore, it is of interest to assess the prevalence, severity, and kinds of post-endodontic periodontal disease (PEP) that emerge in permanent teeth with either necrotic or viable pulp following root canal therapy and following retreatment in paediatric patients.

## Methods and Materials:

## Study population:

This is a prospective study of 127 paediatric patients who had root canal therapy (RCT) for permanent teeth with necrotic pulp vital pulp, or that were previously managed for symptomatic irreversible pulpitis, or whom had the root canal retreatment performed during the course of eight months by a single endodontic specialist.

## Assessment of pulp vitality status:

Only in the cases where the tooth responded to a cold stimulus (CO2 snow) right before treatment and/or there was visible bleeding when the pulp chamber was opened was the pulp status assessed and documented as critical. If the pulp showed no reaction to cold and no signs of bleeding upon opening, it was considered non-vital. A periapical radiographic assessment was used to determine the state of periapical pathology.

## Evaluation of post endodontic pain and use of analgesic drugs 24h postoperatively:

Within 24 hours after surgery, a student (MG), who was not informed of the treatments given, called the patients. She asked them to rate their pain using the same 1-5 point scale they witnessed when they completed the consent form, with 1 being no pain, 2 being light pain, 3 being moderate pain, 4 being severe pain, and 5 being very severe/unbearable agony, six and eighteen hours following treatment. Additionally, patients were asked to describe the kind of pain they were experiencing (spontaneous, triggered by mastication or palpation).

## Statistical analysis:

For evaluating the continuous variables between groups, the independent student's t-test and one- or two-way variance test were employed. To compare the frequencies of categories of variables, the chi-square was employed. In cases where the probability was less than 0.05, differences were deemed significant.

## Results:

The incidence and intensity of PEP in permanent teeth in paediatric patients was greater in teeth in which root canal therapy was conducted in teeth with vital pulp, while it was low in teeth with necrotic pulp at 6 hours and 18 hours after therapy (Table 1). The incidence of spontaneous PEP was greater in all treatment groups as compared to stimulated PEP at 6 hours after treatment. However, incidence of spontaneous PEP was lesser in teeth with vital pulp and teeth with necrotic pulp at 18 hours after treatment. Incidence of spontaneous PEP was however greater in teeth with retreatment at 18 hours after treatment (Table 2).

## Discussion:

It is of interest to assess the prevalence, severity, and kinds of post-endodontic periodontal disease (PEP) that emerge in permanent teeth in paediatric patients with either necrotic or viable pulp following root canal therapy and following retreatment. In our research the incidence and intensity of PEP in permanent teeth in paediatric patients was greater in teeth in which root canal therapy was conducted in teeth vital pulp, while it was low in teeth with necrotic pulp at 6 hours and 18 hours of treatment. This is consistent with a research which found that only twenty-one percent of patients undergoing root canal therapy reported having mild pain, whereas overall fifty-three percent of patients had PEP [12-17]. Some research, however, even among single appointment categories, revealed a decreased frequency [16- 23]. In the current analysis, however, we examined every individual who experienced any amount of PEP earlier studies only involved individuals who had flare-ups. The fact that root canal therapy was completed in a single visit is another element that can account for the greater incidence of PEP in this research. It has been demonstrated that single-visit treatment increases PEP frequency and, in turn, analgesic intake [17, 24- 25].However, without raising short or long term problems, the primary benefits of single-visit therapy are the shortened treatment times and increased patient and dentist convenience [16- 21].There is conflicting evidence in the research literature about the impact of status of pulp-vital or necrotic-on the incidence and degree of severity of PEP [15-19]. According to some studies PEP is more frequently seen after treatment of teeth containing live pulp. These findings are consistent with our own [21-25].

On the other hand, after treating teeth with necrotic pulps some other studies observed a higher incidence of PEP [15- 23]. The disparity could result from disparate endodontic materials and methods or from disparate evaluation criteria for PEP. The results of this research also differ from those of other research that found statistically significant relationships between the frequency of flare-ups following root canal procedures done by residents or students and the existence of periapical lesions [10-24]. The fact that the aforementioned studies only included patients who had flare-ups may contribute to the disparity in this case, as could treatment provided by students or residents. It's unclear why teeth with essential pulp experience a greater degree of occurrence and severity of PEP following therapy [12-15]. One suggestion is that more intense production of mediators of inflammation is encouraged by the damage to periapical vital tissue during endodontic procedures in teeth having vital pulp [11-17].Research has been done, however the results have been conflicting about the relationship between the severity and frequency of flare-ups and the condition of the replaced teeth [9-14]. In contrast to vital teeth, non-vital teeth had flare-ups more frequently after retreatment and endodontic treatment, according to a research. Tooth vitality was found to have no effect on the frequency or severity of flare-ups, according to another research [15- 23]. Compared to other dental operational treatments, pulp therapy and root canal therapy (RCT) cause more frequent and severe postoperative pain, according to data previously released. PEP frequencies fluctuate between 1.5 to fifty-three percent in the literature [12-19]. The wide variation appears to be mostly caused by variations in the definitions of post endodontic discomfort. The majority of research on the frequency of post endodontic pain addressed flare-ups, which were described as excruciating pain and/or oedema following endodontic therapy that necessitated an urgent visit and active therapy [10-16]. Consequently, those studies did not include patients who needed active treatment yet who had discomfort following endodontic treatment [5-11]. One of the most important aspects of endodontic therapy is managing and avoiding the occurrence of PEP. Dentists can improve the opinions of their clients of upcoming dental treatments, raise their pain tolerance, and boost their patients' confidence in their expertise by educating them about expected PEP and offering drugs to lessen it [14, 21]. Studies on the association between the frequency and intensity of flare-ups and the state of the implanted teeth have been conducted, but the findings have been contradictory [16-24]. In the current research, general practitioners managed teeth with clinical irreversible pulpitis by applying Ledermix to the pulp exposure location to reduce dental pain. It is known that the impact of anti-inflammatory drugs on the discomfort associated with teeth of this type [12-19]. A statistically substantial decrease in the occurrence of pain was observed by a research 24 hours after the intracanal anodyne corticosteroid was placed [20- 25]. Data shows higher PEP levels in women are consistent with studies conducted on other populations [12-17]. Gender disparities in pain tolerance could be attributed to variations in men's bodies' responses to pain or to the stereotype that men can handle pain better than women [17-24]. When educating patients about anticipated pain and recommending medications for immediate consumption following endodontic treatment, doctors may find it helpful to pay attention to variations in the incidence and intensity of pain based on pulp state [11-15]. To avoid worsening, alleviating pain should be an essential component of dental treatment, especially in the early phases. When determining whether to prescribe an analgesic, factors like the patient's gender, the number of therapy sessions, and previous encounters with pain as well as analgesics should all be taken into account [16- 19].

## Conclusion:

Root canal therapy of teeth with viable pulp produced a noticeably greater incidence and intensity of PEP in permanent teeth among paediatric patients. Therefore, dentists should to be conscious of this discomfort during management.

## Figures and Tables

**Table 1:** Data regarding incidence and intensity of PEP n different treatment groups at 6 hours and 18 hours after endodontic therapy

		**Vital pulp**	**Necrotic pulp**	**Retreatment**	**P value**
6 hours	Incidence Number (%)	180 (64.9)	40 (39.6)	80 (50.5)	0.003
	Intensity mean ± SD	2.67± 1.5	1.89 ± 1.3	1.91 ± 1.3	0.001
18 hours	Incidence Number (%)	146 (52.9)	36 (35.7)	37 (45.5)	NS
	Intensity mean ± SD	2.11 ± 1.3	1.67 ± 1.1	1.92 ± 1.3	NS

**Table 2:** Data regarding spontaneous PEP and stimulated PEP in different treatment groups at 6 hours and 18 hours after therapy

		**Vital pulp**	**Necrotic pulp**	**Retreatment**
6 hours after treatment	Spontaneous PEP	146 (82.2)	34 (86.1)	64 (81.1)
	Stimulated PEP	34 (19.2)	6 (30)	16 (40)
18 hours after treatment	Spontaneous PEP	46 (45.7)	12(34.4)	44 (60.6)
	Stimulated PEP	82 (56.5)	24 (67.8)	30 (41.6)
